# Role of the anterior insular cortex in restraint-stress induced fear behaviors

**DOI:** 10.1038/s41598-022-10345-2

**Published:** 2022-04-20

**Authors:** Sanggeon Park, Jeiwon Cho, Yeowool Huh

**Affiliations:** 1 Brain and Cognitive Sciences, Scranton College, Ewha Womans University, Seoul, 03760 Republic of Korea; 2 Department of Medical Science, College of Medicine, Catholic Kwandong University, Gangneung-si, 25601 Korea; 3 Ewha Brain Institute, Ewha Womans University, Seoul, 03760 Republic of Korea; 4 Translational Brain Research Center, International St. Mary’s Hospital, Catholic Kwandong University, Incheon, 22711 South Korea

**Keywords:** Emotion, Neural circuits, Stress and resilience

## Abstract

Anxiety disorders, such as post-traumatic stress disorder (PTSD), are thought to occur by dysfunction in the fear and anxiety-related brain circuit, however, the exact mechanisms remain unknown. Recent human studies have shown that the right anterior insular cortex (aIC) activity is positively correlated with the severity of PTSD symptoms. Understanding the role of the aIC in fear and anxiety may provide insights into the etiology of anxiety disorders. We used a modified shock-probe defensive burying behavioral test, which utilizes the natural propensity of rodents to bury potentially dangerous objects, to test the role of aIC in fear. Mice exposed to restraint stress exhibited burying of the restrainer-resembling object, indicative of defensive behavior. Electrolytic ablation of the aIC significantly diminished this defensive burying behavior, suggesting the involvement of the aIC. Single-unit recording of pyramidal neurons in the aIC showed that a proportion of neurons which increased activity in the presence of a restrainer-resembling object was significantly correlated with the defensive burying behavior. This correlation was only present in mice exposed to restraint stress. These results suggest that altered neuronal representation in the aIC may regulate fear and anxiety after exposure to a traumatic event. Overall, our result demonstrates that the aIC mediates fear and anxiety and that it could be a potential target for treating anxiety disorders.

## Introduction

Fear and anxiety are essential for survival^1^, serving a protective and predictive function^2^. These abilities are compromised in patients with anxiety disorders, such as post-traumatic stress disorder (PTSD)^3,4^. Dysfunction of the fear and anxiety-related brain circuits are hypothesized to cause anxiety disorders, however, the exact mechanisms leading to these symptoms remain unknown.

The brain regions involved in regulating fear and anxiety include the hippocampus, medial prefrontal cortex, thalamic nucleus reunions, amygdala, and insular cortex^4^. Among these, the role of the insular cortex in fear is less commonly explored. Nonetheless, results from human studies and extensive inter-connectivity of the insular cortex with other fear and anxiety processing brain regions make it worth extensive investigation. Several human neuroimaging studies have consistently shown that the altered structure and activity of the insular cortex is related to anxiety disorders^5–7^. Additionally, the insular cortex has widespread mutual connections with cognitive and emotional information processing brain regions^8,9^, therefore, it is well positioned to integrate and assess various information. More importantly, the insular cortex is suggested to manage one’s subjective feelings, usually aversive, about various situations^9,10^. Patients with anxiety disorders suffer from subjective personal feelings of fear and anxiety; therefore, understanding how neurons encode these feelings will offer valuable insights into developing better ways to ameliorate debilitating symptoms.

Consistent with human studies, fear conditioning animal studies have shown that the activity of the insular cortex increases with fear^11–13^. In addition, ablations of the insular cortex induced anxiolytic behaviors and disrupted fear memory^14–16^, confirming the involvement of the insular cortex in fear. However, these studies mostly investigated the role of the posterior insular cortex. To the best of our knowledge, no study has revealed the relationship between neuronal activities in the anterior insular cortex (aIC) and fear. A human fMRI study has found that activation of the right aIC, along with the left ventral hippocampus, is positively correlated with the severity of PTSD symptoms^4^. Studying the role of the aIC in fear may provide insight into the etiology of PTSD. To address this, we assessed the relationship between the aIC and fear in mice.

To investigate the role of the aIC in fear, we modified the shock-probe defensive burying test^17–20^, which utilizes the natural propensity of rodents to bury aversive objects. One advantage of the shock-probe burying test is that a single electrical shock makes rodents display defensive behavior (i.e. burying the shock-probe)^17,21,22^, much like a single traumatic event causing PTSD in humans. Instead of using an electrical shock, popularly used in fear conditioning, we used a restraint stress model to mimic a more naturally probable cause of stress because naturalistic experiment models are being acknowledged to better reflect natural functions of the brain^23^. A mice restraint-stress model had been used to reflect PTSD that can develop by being trapped in a narrow space by disasters^24^. Mice that experienced restraint stress exhibited defensive burying behaviors in the presence of a restrainer-resembling object. Using this behavioral model, we further investigated whether the aIC is involved via electrolytical lesioning. We then recorded the activity of individual pyramidal neurons in the right aIC to investigate the detailed neuronal correlates of the behavior.

## Results

### Restraint stress-induced fear behaviors

**Figure 1:**
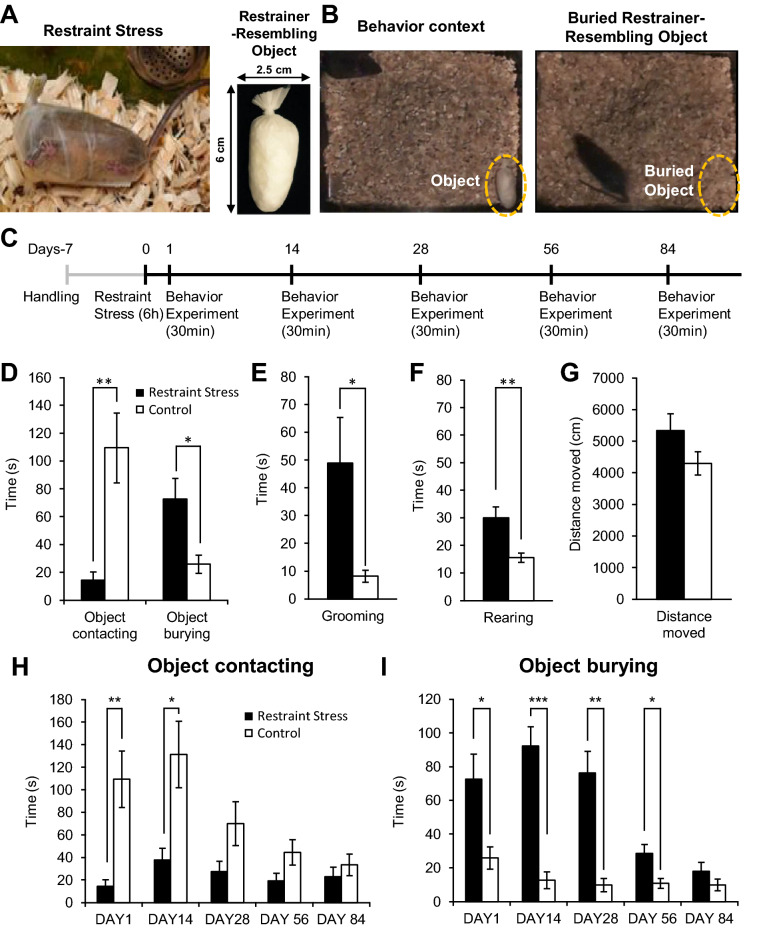
Fear and anxiety-like behaviors induced by acute restraint stress. (**A**) Images of a mouse experiencing restraint stress (left) and a restrainer-resembling object (right). (**B**) Image of an experimental setting (left) and an exemplarily image of a restrainer-resembling object buried by a stressed mouse (right). (**C**) The timeline of experiments. (**D**) Contacting and burying of a restrainer-resembling object in stressed and control mice in the first experiment (day 1). Contacting behavior includes biting, carrying, touching, and manipulation of a restrainer-resembling object. (**E**) Cumulative duration of grooming behavior during in the first experiment (day 1) between groups. (**F**) Rearing behavior (also known as the attempt of escape behavior) of mice in the first experiment (day 1). (**G**) Total distance moved of mice during in the first experiment (day 1). (**H**) Changes in contacting behavior of the restrainer-resembling object over time between stress and control groups. (**I**) Changes in restrainer-resembling object burying behavior over time between stress and control groups. (**D–I**) Control (n = 8), Restraint stress (n = 8). All data are presented as mean ± standard error of mean (SEM). Student’s t-test was used to compare means between the stress and the control group. *p < 0.05, **p < 0.01, ***p < 0.001.

As described in the introduction, we modified the shock-probe defensive burying test by using restraint stress instead of an electrical shock. We measured behavioral differences towards a restrainer-resembling object between mice that experienced a single 6 h restraint stress and mice that did not. Behaviors were measured 24 h after acute restraint stress. Figure 1A shows a representative image of a mouse under restraint stress and a restrainer-resembling object. Sample images of the experimental setting and buried restrainer-resembling object by a restraint-stress experienced mouse are shown in Fig. 1B. The timeline of experimental procedures is outlined in Fig. 1C. As expected, stressed mice significantly reduced contact of the restrainer-resembling object and significantly increased burying time when compared with the control group (Fig. 1D). Stressed mice were initially hesitant to approach a restrainer-resembling object, they started burying after approximately 2 min 28 s and completely buried the object approximately 11 min 45 s after the start of an experiment on average. Coincidentally, the groom and rearing behaviors, respectively corresponding to anxiety-like and exploration-related behaviors in mice, were significantly higher in stressed mice than those of control mice (Fig. 1E, F). Grooming and rearing in stressed mice quickly subsided once the object was buried. Meanwhile, general movement (measured with the total distance moved) did not differ between groups (Fig. 1G), suggesting that locomotion did not affect the behavioral differences between groups. Interestingly, stressed mice displayed these fear and anxiety-like behaviors only in the presence of a restrainer-resembling object, but not in the presence of a neutral object (Supplementary Fig. S1).

The most notable effect of this test is that a single restraint stress was able to produce long-lasting stress-induced fear and anxiety-like behaviors in mice, resembling how PTSD is induced in humans. Surprisingly, mice that experienced restraint stress avoided contacting a restrainer-resembling object for up to 14 days (Fig. 1H). Moreover, the object burying behavior persisted up to the 56th day (Fig. 1I).

**Figure 2:**
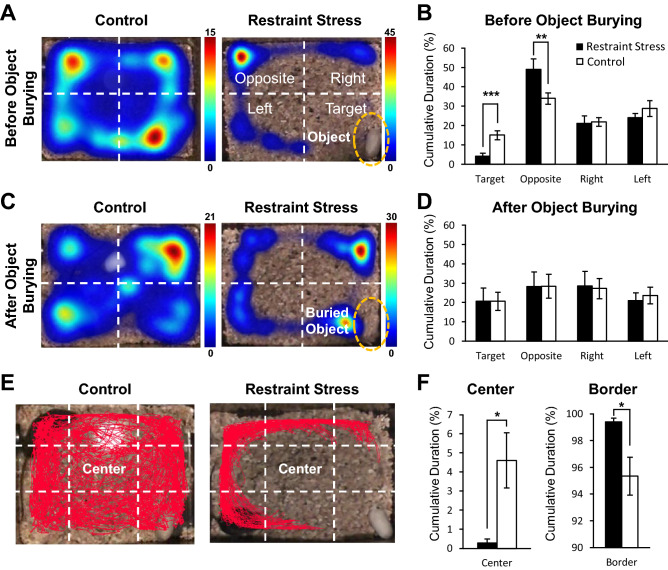
Anxiety-like behavior exhibited in mice exposed to restraint stress. (**A, C**) An experimental cage was divided into four quadrants—opposite (furthest zone from where a restrainer-resembling object was placed), right, left, and target (the zone where a restrainer-resembling object was placed) zones—to analyze differences in movement trajectory between groups. Color scale on the right of each heat map denotes the dwell duration measured in seconds: the hotter the color, the longer the dwell duration. (**A**) Representative heat map analysis showing cumulative dwelling time in the experimental cage of each group before a restrainer-resembling object was buried (0–5 min after experiment). (**B**) Comparison of cumulative duration in respective quadrants between groups before a restrainer-resembling object was buried (0–5 min after experiment). (**C**) Representative heat map analysis showing cumulative dwelling time in the experimental cage of each group (from same animals shown in A) after a restrainer-resembling object was buried (10–15 min after experiment). (**D**) Comparison of cumulative duration in respective quadrants between groups after a restrainer-resembling object was buried (10–15 min after experiment). (**E**) Example trajectory of a control and stressed mice. Red line indicates trajectory of a mouse during the test. White dashed lines divide each experimental cage into the center and border zone. (**F**) Cumulative duration in the center or border zone between groups. (**B, D, F**) All data are presented as mean ± SEM. Student’s t-test was used to compare means between the stress and the control group. *p < 0.05, **p < 0.01, ***p < 0.001. (**A–F**) All the behavioral analyses were performed with data acquired from the first experiment (day 1).

We investigated the behaviors of mice from the first experiment in detail to further scrutinize stress-induced behaviors. First, we divided each test cage into four zones: opposite (furthest zone from where a restrainer-resembling object was placed), right, left, and target (the zone where a restrainer-resembling object was placed) zones (Fig. 2A). Since visibility of a restrainer-resembling object seemed to influence stress-induced behaviors, we compared behaviors from two different experimental time segments: 0–5 min (before object burial) and 10–15 min (after object burial). The representative visit heat map of each group, which shows where and how long a mouse dwelt in each zone, showed that stressed-mouse mostly dwelt in the opposite zone. In contrast, the control mouse moved around all four zones during the 0–5 min of an experiment (Fig. 2A). Accordingly, stressed mice dwelt significantly longer in the opposite zone and significantly shorter in the target zone compared to control mice (Fig. 2B). Interestingly, during the 10–15 min of an experiment, when the restrainer-resembling object is buried and no longer visible, dwelling durations in each zone between the stressed and control mice did not differ (Fig. 2C, D).

Avoidance of the center area is also a measure of anxiety in rodents. Therefore, we analyzed the difference in this behavior between groups. For this analysis, the experimental cage was divided into nine zones (Fig. 2E). Examples of each group’s moving trajectories are depicted in Fig. 2E. Of the nine zones, all eight areas were marked as the border area, excluding the center area. The percentage of dwelling time in either the center or the border area was calculated for each mouse groups. Consistent with the other behaviors analyzed, stressed mice dwelt significantly less in the center zone and significantly more in the border zone compared to control mice (Fig. 2F). These fear and anxiety-like behaviors were absent when a neutral object was present or in absence of any object (no object) (Supplementary Fig. S2).

### Role of the anterior insular cortex in restraint stress-induced fear behaviors

**Figure 3:**
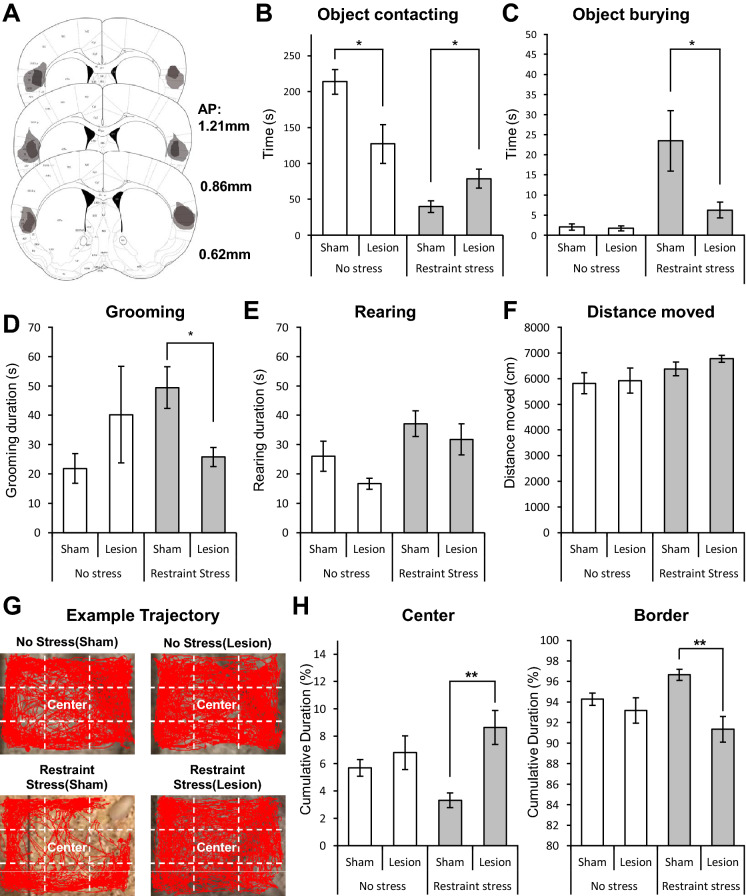
Electrolytic lesion of the aIC in fear and anxiety-like behaviors. (**A**) Histological evaluation of the extent of damage made with electrolytic lesions in a series of sections of the insular cortex. Darker areas indicate complete ablation. Lighter areas indicate partial tissue damage. (**B**) Duration of contacting a restrainer-resembling object in four different groups: no stress (sham) n = 8, no stress (lesion) n = 7, restraint stress (sham) n = 9, and restraint stress (lesion) n = 8. (**C**) Burying of a restrainer-resembling object in different groups (same groups as in **B**). (**D**) Cumulative grooming duration difference between groups. (**E**) Cumulative duration of rearing behavior of different groups. (**F**) Total distance moved in each group (same group in **B**) during the experiment. (**G**) Example trajectory of four different groups. Red lines indicate trajectory of a mouse during the test. White dashed lines show how the center and border zones are defined in each experimental cage. (**H**) Cumulative duration in the center or border zone of different groups. (**B–F, H**) All data are presented as mean ± SEM. Mann–Whitney U test was used to assess statistical difference between groups within the no-stress and the restraint-stress group (*p < 0.05, **p < 0.01).

To investigate the involvement of the aIC in stress-induced fear and anxiety-like behaviors, we compared behavioral differences towards a restrainer-resembling object in stressed mice with [restraint stress (sham)] or without the aIC [restraint stress (lesion)]. The aIC was bilaterally ablated via electrolytic lesions. Lesion locations are depicted in Fig. 3A. Lesioning of the aIC were performed a week before restraint stress, allowing time for recovery. As predicted, fear and anxiety-like behaviors, respectively marked by burying and reduced contacting of the restrainer-resembling object, were present only when the aIC was intact [restraint stress (sham)]. Lesion of the aIC [restraint stress (lesion)] significantly increased contacting duration and significantly decreased burying of the restrainer-resembling object (Fig. 3B, C). Consistently, grooming duration of the restraint stress (sham) mice was significantly higher than the restraint stress (lesion) group (Fig. 3D). The duration of rearing behavior did not differ between sham and lesion restraint stressed group (Fig. 3E), possibly suggesting that the aIC was not involved in stress-induced increase in exploratory behaviors^25^. Total distance moved did not differ between groups (Fig. 3F), suggesting that general movements were not affected by lesion of the aIC. Even though the total distance moved did not differ between groups, the restrain stress (sham) group still had a tendency to avoid the center area relative to the border (Fig. 3G, H), displaying greater anxiety-like behavior than the restraint stress (lesion) group.

We also performed the same experimental protocols in mice groups that did not experience any stress—the no stress (sham) and no stress (lesion)—to examine whether lesioning of the aIC affected any behaviors. We found that, other than the significantly reduced object contacting duration in mice with lesions in the aIC (Fig. 3B), there were no behavioral differences between the no stress sham and lesion groups (Fig. 3C–H).

### Neuronal representation of fear and anxiety in the anterior insular cortex

**Figure 4:**
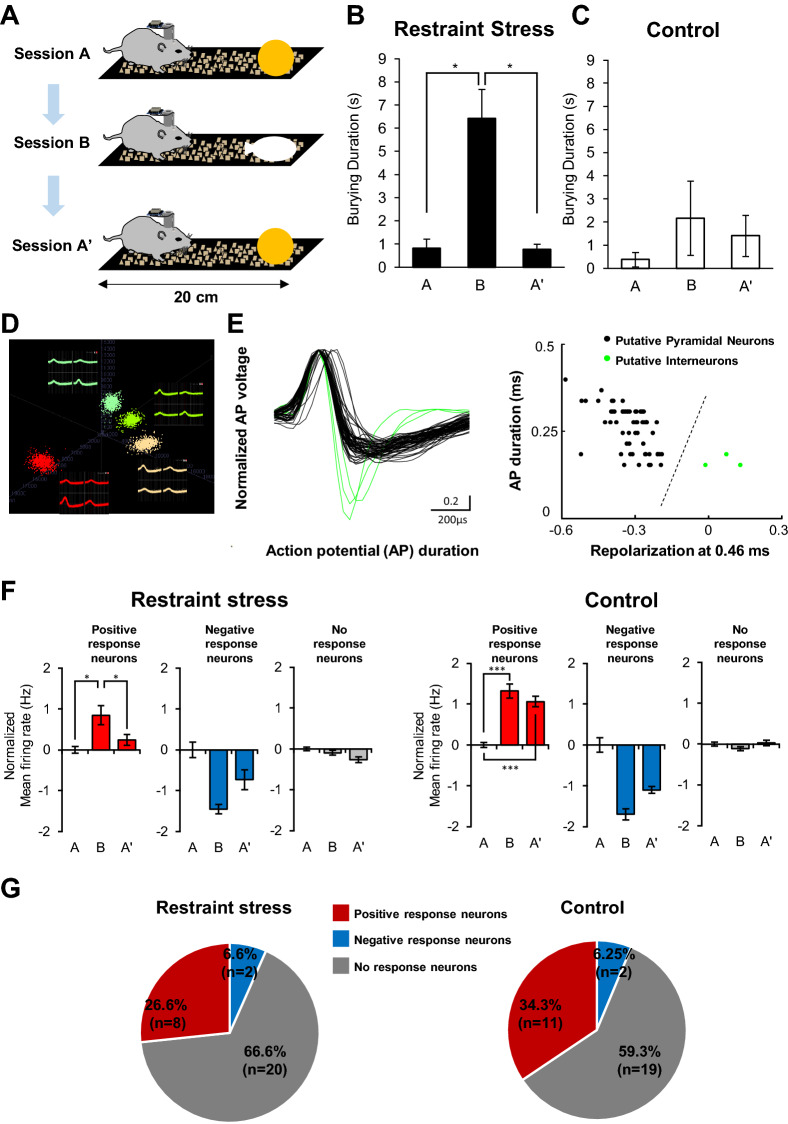
Extracellular single-unit recording of aIC neurons during fear and anxiety-like behavioral assessments. (**A**) Illustration depicting the sequence and experimental setting of behavioral test used for single-unit recording. Mice were presented to a neutral object (session A), restrainer-resembling object (session B), and neutral object (session A’) in sequence. (**B**) Burying duration of mice that experienced restraint stress during the A, B, A’ sessions (n = 5 mice; H(2) = 8.783, p = 0.012, Kruskal–Wallis Test). (**C**) Burying duration of control mice during the A, B, A’ sessions (n = 3 mice; H(2) = 0.655, p = 0.721, Kruskal–Wallis Test). (**D**) Example of a spike-sorting image of isolated single-unit neuronal signals. (**E**) Shapes of recorded action potential (AP) of all individual neurons (left). Each line represents the shape of an action potential of a neuron. Recorded unit neurons were divided into putative-pyramidal neurons or putative-interneurons based on the recorded action potential repolarization time. Putative-pyramidal neurons: black lines or dots. Putative-interneurons: green lines or dots. (**F**) Normalized firing rate of positive (red; stress N = 8, H(2) = 10.343, p = 0.006; control n = 11, H(2) = 22.653, p = 0.001), negative (blue; stress n = 2, H(2) = 4.706, p = 0.095; control n = 2, H(2) = 1.176, p = 0.555), and no-response (gray; stress n = 20, H(2) = 0.642, p = 0.725; control n = 19, H(2) = 2.702, p = 0.259) neurons recorded from restraint stress and control groups during sessions A, B, and A’. Kruskal–Wallis Test was used to test statistical significance of among sessions. (**B**, **C**, **F**) A post-hoc Dunn’s test was used to identify significant differences between sessions (* p < 0.05, ** p < 0.01, *** p < 0.001). (**G**) The relative ratio of positive, negative, and no response neurons in each group. Chi-square tests were used to compare the proportions of response neurons in each group (p = 0.804).

To investigate the neuronal activities in the aIC that correlates with fear and anxiety, we recorded the activity of individual neurons while mice were sequentially exposed to a neutral object (session A), restrainer-resembling object (session B), and neutral object (session A’) (Fig. 4A). The experiment chamber and time were modified due to restrictions imposed by the recording cable. Stressed mice significantly increased the burying duration of the restrainer-resembling object compared with a neutral object (Fig. 4B). This result signifies that the defensive burying measured in our experiment was not due to the propensity of stressed mice to bury novel unfamiliar objects. The burying duration of control mice, on the other hand, was not different between different objects (Fig. 4C). We only used well-isolated single-units in our study, and an example of a spike-sorting image is shown in Fig. 4D. We adapted a method to distinguish putative interneurons from pyramidal neurons from extracellular recordings of neurons in the posterior insular cortex^13^ and divided neuronal types based on recorded action potential shape (Fig. 4E). We only used putative pyramidal neurons in our analysis.

We were interested in neuronal activity changes that occur in the presence of a restrainer-resembling object. We, therefore, normalized neuronal activities based on the average firing rate of neurons in session A. Based on the neuronal activity changes that occurred in session B relative to the activity in session A, we categorized neurons into three groups: positive response (increased firing rate), negative response (decreased firing rate), and no-response neurons (no changes observed). All three types of neurons were present in both the stress and control groups (Fig. 4F). In the presence of a restrainer-resembling object (session B) a proportion of neurons increased firing (stress = 26.6%, control = 34.3%) while few others decreased (stress = 6.6%, control = 6.3%) firing rate (Fig. 4G). Most neurons were no-response neurons with relatively constant firing rates over the sessions in both groups (stress = 66.6%, control = 59.3%). However, when a neutral object was reintroduced (session A’), the firing rates of positive response neurons reverted to the level of session A only in the stressed group. In the control group, the positive and negative response of neurons was sustained in session B and session A’.

### Neuronal activity in the aIC correlates with stress-induced defensive behaviors

**Figure 5:**
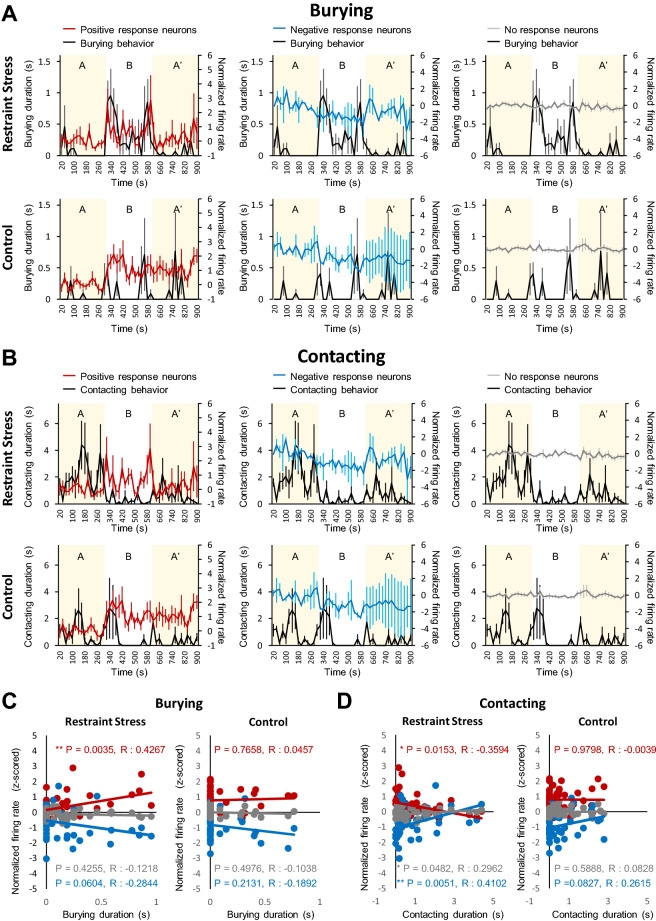
Relationship between fear and anxiety-like behaviors and aIC neuronal activities. (**A**) Normalized average firing rate plotted with the duration of burying behaviors in the restraint stress (top) and control (Bottom) groups. (**B**) Normalized average firing rate plotted with the contacting behavior of a restrainer-resembling object in the restraint stress (top) and control (Bottom) groups. (**C**) Pearson’s correlation between normalized firing rate and the duration of burying behaviors in the restraint stress (left) and control (right) groups. (**D**) Pearson’s correlation between normalized firing rate and the contacting behavior of a restrainer-resembling object in the restraint stress (left) and control (right) groups. (**A–D**) red line: positive response neurons, blue line: negative response neurons, gray line: no response neurons, black line: behaviors (either burying or contacting).

To further investigate the detailed relationship between stress-induced defensive behavior and neuronal activity in the aIC, we analyzed the correlation between behavior and neuronal activities (positive, negative, and no-response neurons) over time (Fig. 5). Interestingly, the activity of positive-response neurons of the stress group had a significant positive correlation, with the burying behavior with an almost time-locked increase in activity when burying behavior occurs (Fig. 5A, C). In addition, there was a strong tendency for the negative-response neurons of the stress group to be negatively correlated with burying behaviors (p = 0.06). However, none of the neuronal activities were correlated with burying behaviors in the control group, although they sporadically displayed burying behaviors (Fig. 5A, C). The results suggest that some neuronal activities in the aIC correlates with defensive behavior only when animals experience stress.

Reduced contacting behaviors in the stress group showed a significant correlation with neuronal activities (Fig. 5B). In the stress group, positive-response neurons had a negative correlation, while negative-response neurons had a positive correlation with burying behaviors (Fig. 5D). In the control group, however, no neuronal activities were correlated with contacting behaviors (Fig. 5D). In any case, none of the no-response neurons was correlated to any behaviors assessed in neither the stress nor the control group (Fig. 5A–D). Overall, our analysis demonstrates that only a subset of neurons in the aIC changes activity in accordance with defensive behaviors in mice that had experienced restraint stress. While control mice have positive and negative-response neurons in the aIC, none of their activities were related to burying or contacting behaviors.

## Discussion

Our study suggests that the aIC could be an important brain region in mediating aversive behaviors in mice after exposure to a traumatic event. We first demonstrated that a single restraint-stress was sufficient to trigger fear and anxiety-like behaviors towards a restrainer-resembling object by measuring contacting and burying behaviors. Surprisingly, these behaviors lasted for a long time, suggesting that a single memorable aversive event could have a lasting behavioral effect in animal models. We then found that electrolytic ablation of the aIC significantly reduced these fear and anxiety-like behaviors to the level of no stress mice, confirming the importance of the aIC in these behaviors. Furthermore, we found that the activity of some pyramidal neurons in the aIC was significantly correlated with defensive behaviors only in mice that experienced stress.

Fear and anxiety are important for survival^1^. Fear helps us flee from imminent danger, while anxiety helps us prepare for dangers that may occur in the future. Pathological disorders may occur when the fear and anxiety-related brain circuits become dysfunctional, no longer serving their useful function. Our animal model does not reflect pathological stress-induced behaviors, however, it still offers an additional animal model to understand how PTSD may be mediated. With further development and validation, our animal model may be useful for studying how fear and anxiety generalization occurs. Generalization is a fear response triggered by stimuli that are partially similar to the original stimulation^26^. It is a main symptom of anxiety disorders. In our animal model, a restrainer-resembling object, which is not the actual restrainer, triggered fear and anxiety-like behaviors, which resembles generalization. Failure of mice to perceptually discriminate a restrainer from a restrainer-resembling object could also induce these behaviors; therefore, this aspect needs to be validated before our model is used to study generalization. Our animal model could also offer insight into understanding the mechanisms of resilience to stressful events, since burying behavior is suggested to be an active coping mechanism of stress^17,19^. Studying the burying behavior of mice could offer insight into understanding the mechanisms of resilience to stressful events.

The results of our aIC lesion study support that the aIC is necessary to acquire restraint stress-induced aversive behaviors towards a restrainer-resembling object. With lesion in the aIC, fear and anxiety-like behaviors—defensive burying, reduced contacting of a restrainer-resembling object, increased grooming, reduced duration in the center area—significantly reduced. Behavioral differences between the sham and aIC lesion group are unlikely due to the effect of aIC lesion, because no behaviors, except for object contacting, differed between the sham and lesion group that did not experience stress. Reduced object contacting behavior in the no stress (lesion) group may have occurred due to a reduced ability to recognize objects (the insular cortex is known to be involved in object recognition)^27^. Even with the possible reduction in the ability to recognize objects by aIC lesion, between groups that experienced stress, the sham group had significantly lower object contacting duration than the lesion group. This suggests that the aIC is involved in mediating this stress-induced behavior. By contrast, rearing behavior was increased in mice that experienced restraint-stress regardless of absence or presence of the aIC. This suggests that stress-induced exploratory behavior may not be influenced by the aIC because rearing behavior is suggested to be a measure of inspective and diversive exploratory behavior in mice^25^. Taken together, behavioral results suggest that the aIC is necessary for acquiring or expressing fear and anxiety-like behaviors but not in regulating exploratory behavior.

Single-unit recording analysis of pyramidal neurons in the aIC showed that the positive and negative-response neurons had a significant correlation with fear and anxiety-like behaviors in the stressed group. The positive and negative-response neurons were categorized based on neuronal activity changes to object replacement between sessions—A, B, and A’—in reference to session A, so the activity of these neurons will reflect changes induced by changing objects in sessions B and A’. In the stress group, however, the changing activity of positive and negative-response neurons in the aIC was correlated with fear and anxiety-like behaviors such as burying and contact avoidance of a restrainer-resembling object in session B. This could mean that exposure to stress may have shifted the salience circuit to react primarily to an object that recurs the experience since the aIC, among subdivision of the insular cortex, is suggested to be a key node in salience processing^28^. The positive and negative-response neurons in the control group may be a default mode responding to changes in objects or environment because their changes in neuronal activity were maintained in both sessions B and A’, and the insular cortex is known to be involved in object recognition^27^. These neurons may obtain valence that links fear and anxiety-like behavior after exposure to stress because none of the activity of these neurons in the control group had any correlation with contacting or burying behaviors, even though mice in the control group do display the behaviors occasionally.

Similar to the aIC neurons found in our study, the posterior insular cortex (pIC) had neurons that respond both positively and negatively to a conditioned stimulus^13^. The exact relationship between positively and negatively responding neurons in the aIC and pIC remains elusive at this point, however, they would likely play distinctive roles. The aIC and pIC have different connectivity patterns that may confer each brain region with different roles; the aIC has connections with brain regions processing high order cognition and emotion, such as the medial prefrontal cortex and anterior cingulate cortex, while the pIC has connections with brain regions processing sensory information^8^. Agranular cortices were predicted to be apt for making assessments and predictions^29^, and the aIC, being an agranular cortex^8^, may serve those functions by obtaining sensory information through the pIC and integrating information from other brain regions. Disorders like PTSD may arise in part from a dysfunctional prediction and error assessment mechanism in the aIC. Therefore, the aIC may be a potential target for regulating stress-induced maladaptive behaviors.

## Methods

### Animals

All experiments were approved and conducted following the guidelines of the Institutional Animal Care and Use Committee (IACUC) of Ewha Womans University (EWHA IACUC 21-008-t). Our research is in accordance with ARRIVE 2.0 guidelines^30^. F1 hybrids of C57BL/6 J × 129/SvJae male mice were used in all experiments. Mice were kept at constant temperature (23 ± 1 °C) and humidity (50 ± 5%) with free access to food and water in an alternating 12-h dark–light reversal cycle. For all experiments, mice were initially group caged then individually caged after handling for single-unit recording and behavioral assessments.

### Acute restraint stress

After seven consecutive days of handling for 10 min, mice in the restraint stress group received acute restraint stress (6 h) using the thumb part of a medium-size synthetic vinyl glove (Dae Myung Science, South Korea) (Fig. 1A), and left in a clean cage with aspen shaving, identical to their home cage. A small pore was made around the nose of a mouse to allow the mouse to breathe and wrapped around with biaxially oriented polypropylene tape throughout the body and tied with cable ties around the tail to prevent escape. Polypropylene tape has an acrylic adhesive that smells like strong vinegar. Extra care was given to apply consistent moderate pressure among mice. After restraint stress, mice were released from the restrainer and stayed with the restrainer for an extra hour. Control mice underwent the same procedures without the restraint stress, staying in the same room as the stress group a clean cage and bedding identical to their home cages.

### Behavior tests and analysis

Defensive burying behaviors were measured at 24 h, day 14, day 28, day 56, and day 84 after the acute restraint stress session. Restraint stress session and behavioral tests were carried out in different rooms. Behaviors of mice in the presence of a restrainer-resembling objection, made of the thumb part of a medium-size synthetic vinyl glove (Dae Myung Science, South Korea) stuffed with tissues (Fig. 1A), were recorded for 30 min using a video camcorder (Sony, HDR-CX560) (Fig. 1B). Mice that did not receive restraint stress were used as control and underwent the same behavioral test. Tests were performed in cages used for home cages, but not their home cage, filled with different bedding (chip bedding) to minimize an unfamiliar environment affecting experimental results (Fig. 1B). Video recordings were manually analyzed by experienced researchers blinded to the test groups. The duration of burying or contacting of the restrainer-resembling object, and grooming and rearing behaviors were manually analyzed. Burying behavior was scored when mice showed shoving of bedding material with its forepaws towards an object, building up bedding, and eventually covering the object. Contacting behavior was scored when mice physically touched the object. Grooming behavior was scored when mice showed any grooming behavior performed on themselves (licking, scratching, and nibbling). Rearing behavior was scored when mice stood up in their rear limbs. The total duration mice participated in each behavior was summed up for each mouse and then averaged. Moving trajectory heat map analysis, moved trajectory plotting, center versus border dwelling percentage, and total distance mice moved were plotted and calculated using EthoVisionXT (Noldus, Wageningen, Netherlands). For center and border dwelling time calculation, the percentage of dwelling time in the center or border (all zones excluding the center zone) was calculated for each mouse then averaged.

### Electrolytic lesion of the aIC

To investigate the role of aIC in generalized behaviors, mice underwent stereotaxic surgery to make bilateral electrolytic lesions in the aIC. After placing a custom-made unipolar lesion electrode (26AWG, copper wire) in the aIC (AP: + 1.2 mm, ML: ± 3.4 mm, DV: −1.8 mm) anodal constant direct current (0.5 mA for 10 s) was delivered in each hemisphere. Sham surgery control groups underwent the same surgical procedure—inserting and removing a lesion electrode in the same aIC coordination as the lesion groups—without current delivery. Half of the lesion and sham surgery control groups underwent acute restraint stress (6 h), and the other half did not experience any restraint stress. All mice were allowed to recover from surgery for 3 days and then handled for a week before the experiment. Same experimental procedures and some analysis methods used in the non-lesioned animals were used.

### Extracellular single-unit recording in behaving mice

#### Surgery

Mice were anesthetized with Zoletil (30 mg/kg, i.p.) and placed on a stereotaxic instrument (David Kopf Instruments, USA) for surgery. Craniotomy was performed above the target region to implant a Microdrive equipped with four tetrodes (Four nichrome polyamide-insulated microwires of 12.5 μm intertwined into one tetrode, Kanthal precision technology; recording tip of each tetrode channel was gold plated to 300–400 kΩ measured at 1 kHz, Bak Electronics). After implanting tetrodes in the anterior insular cortex (AP: + 1.2 mm, ML: −3.4 mm, DV: −1.8 mm; right hemisphere) the Microdrive was secured onto the skull with self-tapping stainless-steel screws and dental cement (Vertex dental, Netherlands). Mice were allowed to recover from surgery for 3 days and then were handled for 7 consecutive days (5 min each day).

#### Single-unit recording

All single-unit recording sessions started after recovery and handling. Neural activities were amplified (10,000×), filtered (600 Hz to 6 kHz), and digitized (30.3 kHz) using a Digital Lynx (Neuralynx, Tuscon, AZ) to obtain unit signal of neurons. Upon successful identification of unit signals, mice had an hour break in their home cages. Afterward, mice in the restraint stress group underwent acute restraint (6 h) in a separate cage with new bedding which is identical to the ones used in home cages. The next day, unit activity of aIC neurons was recorded in freely behaving mice during three sessions in a 20 cm long black linear chamber with chipped bedding, which is different from the ones used in home cages. Mice were sequentially introduced to a neutral object (Orange ping-pong ball; session A), a restrainer-resembling object (restrainer-resembling object; session B), and a neutral object (Orange ping-pong ball; session A’), 5 min per session. Mice were briefly placed in a square black polycarbonate box (20 × 23 × 15 cm) between sessions, while objects were manually exchanged. Behavioral videos and extracellular single-unit recordings signals were simultaneously acquired with a Digital Lynx (Neuralynx, Tuscon, AZ), which allows video and neural signal synchronization.

#### Spike data analysis

Single-units manually isolated using the Spike Sort 3D (Neuralynx, USA) program. Cluster quality of each isolated unit data will be assessed by L-ratio (L-ratio < 0.05), cross-correlation, and inter-spike interval (ISI) in the ISI histogram (ISI > 1 ms)^31^. Only well-isolated unit data were used in our analysis. Isolated single-unit signals of the IC neurons were further classified as putative pyramidal neurons or interneurons. Putative interneurons have short repolarization and duration of action potentials compared with putative pyramidal neurons (Fig. 2A, B)^13^. In this study, we only used putative pyramidal neurons in the aIC for analysis.

Firing rates of putative pyramidal neurons were normalized using the z-scored method that uses the average firing rate of session A using the following equation (*value* = firing rate at a given time bin, μ = average firing rate session A, σ = standard deviation of session A firing rate). Bin size of neuronal signal analysis was either 5 min (Fig. 4) or 20 s (Fig. 5).

$$z= \frac{value-\mu }{\upsigma }$$

Units were classified as either positive-response or negative-response neurons if their z-score in session B was above or below 1.96 standard deviations (SD), respectively, like analysis reported elsewhere^13,32,33^. Units neither classified as positive-response nor negative-response neurons have been classified to be no-response neurons. Single-unit activities were processed and analyzed using a custom MATLAB (R2019b) script.

#### Relationship analysis between behavior and neuronal activities

Pearson’s correlation coefficients were computed between behaviors and each type of neuronal activities analyzed in 20 s time bins. p < 0.05 was determined the level of significance.

### Histology

Locations of the neuronal signal recording verified with histology. After overdosing mice with 2% avertin and small electrolytic lesions were made at the tip of the recording site bypassing anodic current (10 µA, 5 s) through tetrode channels. Then mice were trans-cardially perfused with saline (0.9%) followed by formalin (10% formalin diluted in saline). Brains were removed and stored in formalin (10% formalin diluted with deionized water) for a day and transferred to a 30% sucrose solution for another day for further fixation. Fixed brain tissues were frozen cut in coronal sections (50 µm-thick) through the anterior insular cortex with a microtome (HM525, Microm). Brain slices were placed on top of microscope slides, dried for a day, and stained with cresyl violet (Sigma, USA). Stained slides were examined under a light microscope to determine the locations of the electrolytic lesion.

### Statistical analysis

The student’s t-test was used to compare the means of all behavior assessments between restraint stress and control groups for parametric data. The Mann–Whitney U Test was used to compare groups for non-parametric data. To investigate the long-lasting effects of acute restraint stress, the one-way repeated-measures ANOVA with Bonferroni post-hoc analysis was used to compare means between groups. The Kruskal–Wallis test with Dunn’s test post hoc analysis was used to compare means between groups or sessions that were not normally distributed. The Chi-square test was used to compare differences in the proportion of neuronal response types between the stressed and control groups. All statistical analyses were performed with the SPSS 26.0 (SPSS Inc., USA).

## Supplementary Information

Supplementary Figures.

## Data Availability

The datasets used in the current study will be available from the corresponding author on reasonable request.
