# Supplementary material for: Role of the anterior insular cortex in restraint-stress induced fear behaviors
Source: Sci Rep. 2022 Apr 20;12:6504. doi: 10.1038/s41598-022-10345-2 (PMC9021273; doi:10.1038/s41598-022-10345-2)
Supplement: Supplementary file 1 — Supplementary Figures. [file 41598_2022_10345_MOESM1_ESM.docx]

Supplementary materials for :

**Role of the anterior insular cortex in restraint stress-induced fear behaviors**

Sanggeon Park, Jeiwon Cho, Yeowool Huh

Corresponding author: Jeiwon Cho ([jelectro21@ewha.ac.kr](mailto:jelectro21@ewha.ac.kr)) and Yeowool Huh ([huh06@cku.ac.kr](mailto:huh06@cku.ac.kr))


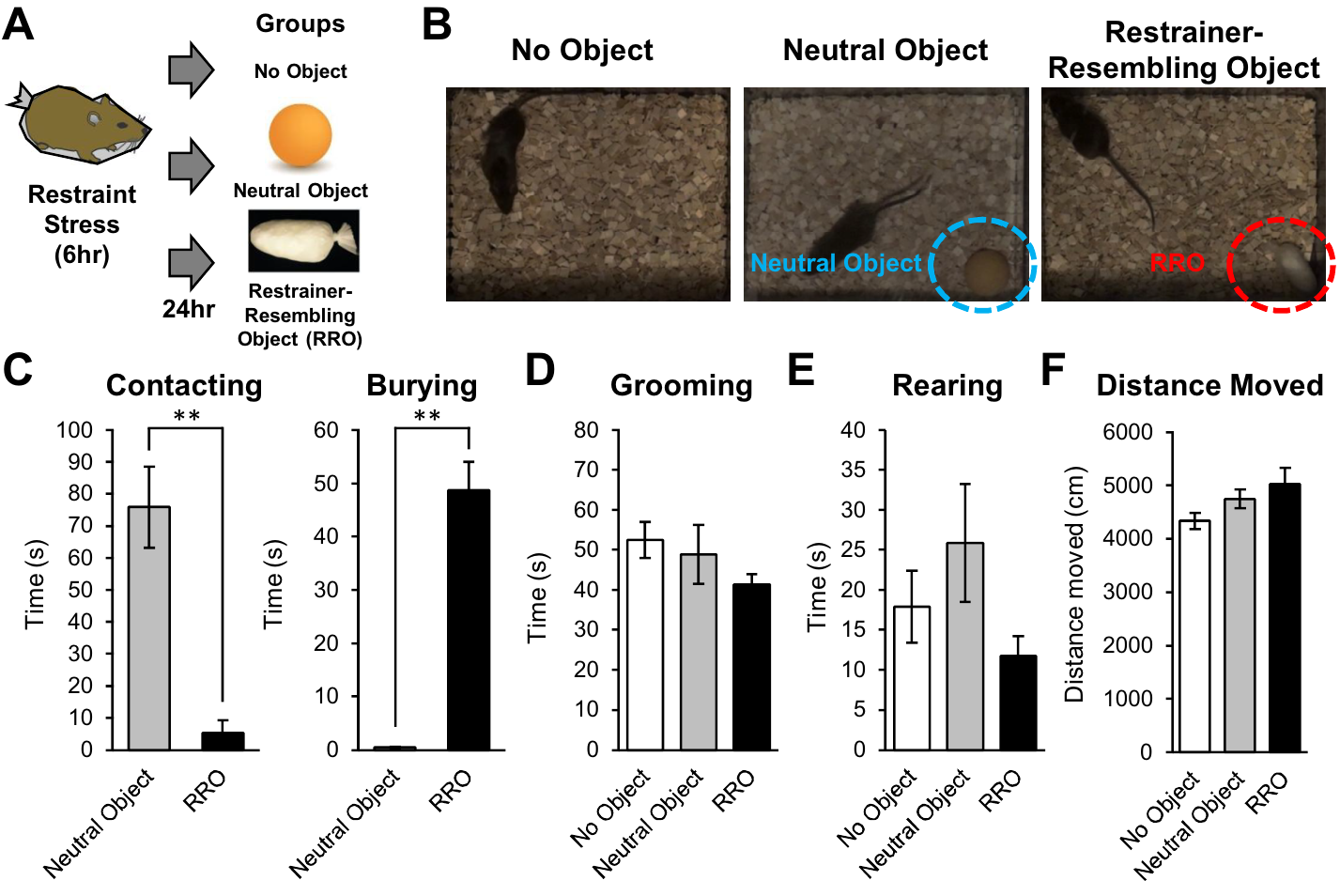


**Figure S1.**

(**A**) Illustration of the experimental procedures. After restraint stress behaviors in three different conditions were tested. Groups tested: no object n=6, neutral object n=6, and restrainer-resembling object n=8. (**B**) Image of an experimental setting of no object (left), neutral object (middle), and restrainer-resembling object (right). (**C**) Cumulative contacting and burying duration of mice exposed to a neutral object or a restrainer-resembling object. Mann-Whitney Test was used to test statistical significance between groups. ** p<0.01. (**D**) Cumulative duration of grooming behavior of different groups (H(2)=1.542, p=0.463, Kruskal-Wallis Test). (**E**) Cumulative duration of rearing behavior of different groups (H(2)=6.246, p=0.044, Kruskal-Wallis Test). (**F**) Total distance moved in each group during the experiment (H(2)=4.262, p=0.119, Kruskal-Wallis Test). (**C-F**) Data are presented as mean ± standard error of mean (SEM).


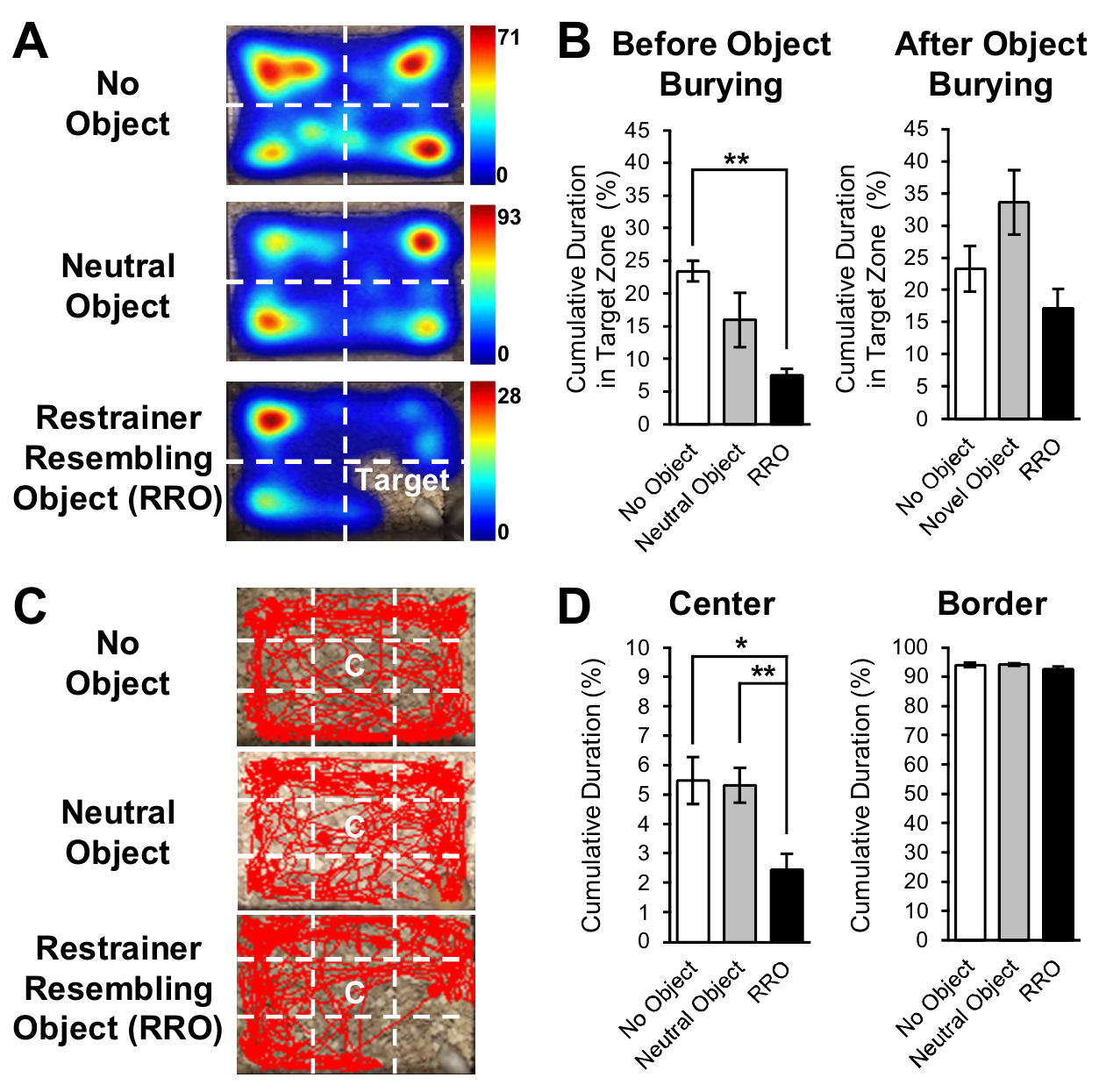


Figure S2.

(**A**) Representative heat map analysis showing cumulative dwelling time in the experimental cage of each group before a restrainer-resembling object was buried (0-5 min after experiment). (**B**) Comparison of cumulative duration in target zone between groups before a restrainer-resembling object was buried (0-5 min after experiment; H(2)=9.581, p=0.008, Kruskal-Wallis Test) and after a restrainer-resembling object was buried (10-15 min after experiment; H(2)=4.190, p=0.123, Kruskal-Wallis Test). (**C**) Example trajectories of each group. Red line indicates the trajectory of a mouse during the test. White dashed lines divide each experimental cage into the center and border zone. (**D**) Cumulative duration in the center or border zone of each groups (Center, H(2)=7.408, p=0.025; Border, H(2)=1.637, p=0.441, Kruskal-Wallis Test). (**B** and **D**) All data are presented as mean ± SEM. Dunn’s test post-hoc was used for comparison between groups. * p<0.05, ** p<0.01.
